# Complete mitochondrial genomes of two water mite species: *Hygrobates* (*H.*) *longiporus* and *Hygrobates* (*rivobates*) *taniguchii* (Acari, Trombidiformes, Hygrobatoidea)

**DOI:** 10.1080/23802359.2020.1793226

**Published:** 2020-07-23

**Authors:** Shimpei F. Hiruta, Shizuko Morimoto, Gyo Yoshinari, Tom Goldschmidt, Kanto Nishikawa, Satoshi Shimano

**Affiliations:** a Center for Molecular Biodiversity Research, National Museum of Nature and Science, Tsukuba, Japan; b Museum of Nature and Human Activities, Sanda, Hyogo, Japan; c Idea Consultants Inc., Yaizu, Shizuoka, Japan; d Zoologische Staatssammlung München, München, Germany; e Graduate School of Global Environmental Studies, Kyoto University, Kyoto, Japan; f Graduate School of Human and Environmental Studies, Kyoto University, Kyoto, Japan; g Science Research Center, Hosei University, Tokyo, Japan

**Keywords:** Trombidiformes, *Hygrobates*, *Hygrobatoidea*, water mite, complete mitogenome

## Abstract

We determined the complete mitochondrial genome sequences of two water mites, *Hygrobates* (*Hygrobates*) *longiporus* and *H.* (*Rivobates*) *taniguchii*. The length of the entire mitogenome of these two species is 13,721 bp and 13,770 bp long, respectively. Both of them contain 13 proteins, two rRNAs, and 22 tRNAs for a total of 37 gene products. The gene order of *Hygrobates* is almost identical to the two species of *Unionicola* we included in the analysis, with some difference in the position of transfer RNA genes. Phylogenetic analyses highly support these *Hygrobates* species form a clade with other species of the Hygrobatoidea.

Diverse assemblages of water mites are present in almost all freshwater environments around the world except Antarctica. The diversity of the water mites is now rapidly becoming apparent, with over 10,000 species estimated to be present (Di Sabatino et al. 2008), while approximately 7,500 species have been described so far (Smit in press). Recently as well as molecular research on water mites greatly developed; molecular studies showed the monophyly of the Hydrachnidia as well as all but one of the traditional superfamilies (Dabert et al. 2016). In several studies, cryptic species, as well as species complexes, have been detected (Martin et al. 2010; Pešić & Smit 2016, 2017, 2018; Pešić et al. 2017, 2019; Blattner et al. 2019). Water mites reach great abundance and diversity in several freshwater environments and are associated with a variety of macrofauna––adults and deutonymphs of most species are predators mainly on microcrustaceans and aquatic insect eggs and larvae (one group has been found to parasitize amphibians (Goldschmidt et al. 2020)) but the larvae are parasites of aquatic insects. Therefore, it has been noted that they are excellent bioindicators (Goldschmidt 2016). On the contrary, their diversity is greatly underestimated, suggesting the presence of multiple cryptic species within many morphological species (Pešić et al. 2017; Blattner et al. 2019).

Thus, more genetic information is urgent to elucidate phylogenetic relationships and genetic structure for revising taxonomy and species diversity in water mites. However, there was no mitogenome record for the family Hygrobatidae, one of the major families in water mites. So, we choose two *Hygrobates* species for the representative of the family and determined the whole mitogenome sequences by shotgun sequencing for both species.

Total DNA was extracted using DNeasy Blood & Tissue Kit (QIAGEN, Hilden, Germany) and processed by QIAseq FX DNA Library kit (QIAGEN). Paired-end sequencing (300 cycles) was conducted by National Museum of Nature and Science, Tokyo on MiSeq, with inserts of ca. 50–200 bp, for a total of ca. four million reads. Assembly was performed using CLC Genomics Workbench ver. 12 (QIAGEN) with default setting. Ambiguous part of the contig was proofread by 3500 xL Genetic Analyzer (Thermo Fisher Scientific, Waltham, MA, USA). Gene identification was done using MITOS web server (Bernt et al. 2013). Voucher specimens with extracted DNA were deposited to National Museum of Nature and Science, Tokyo (NSMT-DNA 49380 and 49381).

The entire mitogenome of *Hygrobates* (*Hygrobates*) *longiporus* Thor, 1898 (GenBank/DDBJ/EMBL accession number LC552026) and *Hygrobates* (*Rivobates*) *taniguchii* Imamura, 1954 (LC552027) is 13,721 bp and 13,770 bp long, respectively. Both of them contain 13 proteins, two rRNAs, and 22 tRNAs for a total of 37 gene products. The overall A + T content of the *H.* (*H.*) *longiporus* and *H.* (*R*.) *taniguchii* mitochondrial genome is 72.5 and 67.9%, respectively, which is in ordinal range among Parasitengona species (67.5–73.6%). In the mitogenomes of both species, ND5 starts with CTT codon and stops with incomplete termination condon T. The gene order of *Hygrobates* is almost identical to the species of genus *Unionicola*, with some difference in the position of transfer RNA genes.

The maximum-likelihood phylogenetic analysis (ML) based on 13 protein coding genes was conducted by RAxML-NG ver.0.9.0 (Kozlov et al. 2019) with bootstrap analyses of 1,000 replicates. The phylogenetic tree also with posterior probability from Bayesian analyses (BA) conducted by MrBayes 3.2.6 (Ronquist et al. 2012). *H.* (*H.*) *longiporus* and *H.* (*R.*) *taniguchii*. made a sister clade with species belonging to superfamily Hygrobatoidea (*Unionicola parkeri* and *U. foili*) with high nodal support value (Figure 1). These mitogenomes would be useful for reconstructing higher systematics of Hydrachnid mites.

**Figure 1.**
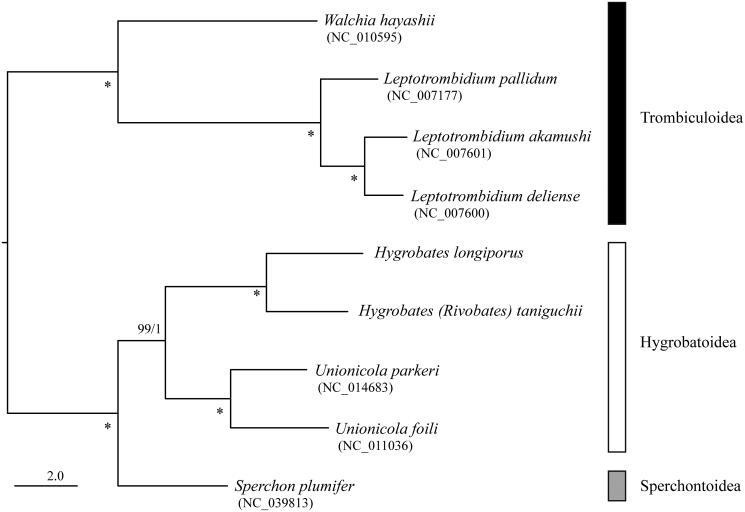
Maximum-likelihood tree based on the concatenated nucleotide sequence of 13 protein-coding genes of *Hygrobates (Hygrobates) longiporus* (LC552026) and *Hygrobates* (*Rivobates*) *taniguchii* (LC552027), three further Hydrachnidia and four Trombiculoidea species. Accession numbers of the mitogenome sequences for each taxon used in the phylogenetic analysis are shown in parentheses. Nodal values are ML bootstrap support values (BS) and BA posterior probabilities (PP). An asterisk (*) indicates 100% BS and 1.0 PP. The scale bar indicates branch length in substitutions per site.

## Data Availability

The data that support the findings of this study are openly available in the National Center for Biotechnology Information database (NCBI/GenBank) at https://www.ncbi.nlm.nih.gov/, accession numbers LC552026 and LC552027.

## References

[CIT0001] Bernt M, Donath A, Jühling F, Externbrink F, Florentz C, Fritzsch G, Pütz J, Middendorf M, Stadler PF. 2013. MITOS: improved *de novo* metazoan mitochondrial genome annotation. Mol Phylogenet Evol. 69(2):313–319. 22982435 10.1016/j.ympev.2012.08.023

[CIT0002] Blattner L, Gerecke R, von Fumetti S. 2019. Hidden biodiversity revealed by integrated morphology and genetic species delimitation of spring dwelling water mite species (Acari, Parasitengona: Hydrachnidia). Parasit Vectors. 12(1):492. 31639027 10.1186/s13071-019-3750-y PMC6805402

[CIT0003] Dabert M, Proctor H, Dabert J. 2016. Higher-level molecular phylogeny of the water mites (Acariformes: Prostigmata: Parasitengonina: Hydrachnidiae). Mol Phylogenet Evol. 101:75–90. 27150348 10.1016/j.ympev.2016.05.004

[CIT0004] Di Sabatino A, Smit H, Gerecke R, Goldschmidt T, Matsumoto N, Cicolani B. 2008. Global diversity of water mites (Acari, Hydrachnidia; Arachnida) in freshwater. Hydrobiologia. 595(1):303–315.

[CIT0005] Goldschmidt T, Nishikawa K, Hiruta SF, Shimano S. 2020. Description of three new water mite species of *Hygrobates* Koch, 1837 (Lurchibates Goldschmidt & Fu, 2011) (Acari. Hydrachnidia, Hygrobatidae), Parasitic in Newts of the Genera Paramesotriton and Pachytriton (Amphibia, Caudata, Salamandridae) from China. Zootaxa. 4768(1):25–42. 10.11646/zootaxa.4768.1.3 33056534

[CIT0006] Goldschmidt T. 2016. Water mites (Acari, Hydrachnidia): powerful but widely neglected bioindicators–a review. Neotrop Biodiv. 2(1):12–25.

[CIT0007] Kozlov A, Darriba D, Flouri T, Morel B, Stamatakis A. 2019. RAxML-NG: A fast, scalable and user-friendly tool for maximum likelihood phylogenetic inference. Bioinformatics. 35(21):4453–4455. 31070718 10.1093/bioinformatics/btz305 PMC6821337

[CIT0008] Martin P, Dabert M, Dabert J. 2010. Molecular evidence for species separation in the water mite *Hygrobates nigromaculatus* Lebert, 1879 (Acari, Hydrachnidia): evolutionary consequences of the loss of larval parasitism. Aquat Sci. 72(3):347–360.

[CIT0009] Pešić V, Asadi M, Cimpean M, Dabert M, Esen Y, Gerecke R, Martin P, Savić A, Smit H, Stur E. 2017. Six species in one: evidence of cryptic speciation in the *Hygrobates fluviatilis* complex (Acariformes, Hydrachnidia, Hygrobatidae). SAA. 22(9):1327–1377.

[CIT0010] Pešić V, Saboori A, Zawal A, Dabert M. 2019. Hidden but not enough: DNA barcodes reveal two new species in *Hygrobates fluviatilis* complex from Iran (Acariformes, Hydrachnidia, Hygrobatidae). Syst App Acarol. 24(10):2439–2459.

[CIT0011] Pešić V, Smit H. 2016. Evidence of cryptic and pseudocryptic speciation in *Brachypodopsis baumi* species complex (Acari, Hydrachnidia, Aturidae) from Borneo, with description of three new species. Syst App Acarol. 21(8):1092–1106.

[CIT0012] Pešić V, Smit H. 2017. *Neumania kyrgyzica* sp. nov. a new water mite from Kyrgyzstan based on morphological and molecular data (Acari, Hydrachnidia: Unionicolidae). SAA. 22(6):885–894.

[CIT0013] Pešić V, Smit H. 2018. A second Palaearctic species of the genus *Wettina* Piersig, 1892 based on morphological and molecular data (Acari, Hydrachnidia: Wettinidae). Syst App Acarol. 23(4):724–732.

[CIT0014] Ronquist F, Teslenko M, Van Der Mark P, Ayres DL, Darling A, Höhna S, Larget B, Liu L, Suchard MA, Huelsenbeck JP. 2012. MrBayes 3.2: efficient Bayesian phylogenetic inference and model choice across a large model space. Syst Biol. 61(3):539–542. 22357727 10.1093/sysbio/sys029 PMC3329765

[CIT0015] Smit H. In press. Water mites of the world, with keys to the families, subfamilies, genera and subgenera (Acari: Hydrachnidia). Dutch Entomological Society, Amsterdam, Netherlands.

